# Effects of Arbuscular Mycorrhizal Fungi and Metal-Tolerant *Pseudomonas fluorescens* on Mitigating Cadmium and Zinc Stress in Tomato

**DOI:** 10.3390/plants14213353

**Published:** 2025-10-31

**Authors:** Leilei Zhang, Gabriele Bellotti, Hajar Salehi, Edoardo Puglisi, Luigi Lucini

**Affiliations:** 1 Department for Sustainable Food Process, Università Cattolica del Sacro Cuore, 29122 Piacenza, Italy; gabriele.bellotti@unicatt.it (G.B.); hajar.salehi@unicatt.it (H.S.); edoardo.puglisi@unicatt.it (E.P.); luigi.lucini@unicatt.it (L.L.); 2 Institute of Bioimaging and Complex Biological Systems (IBSBC), National Research Council (CNR), 20054 Milan, Italy

**Keywords:** rhizosphere microbiome, multi-omics, bioremediation, microbial biostimulants, abiotic stress tolerance, metabolomics

## Abstract

Heavy metal (HM) contamination in agricultural soils poses a significant threat to soil health and plant productivity. This study investigates the impact of cadmium (Cd) and zinc (Zn) stress on tomato plants (*Solanum lycopersicum*) and explores the mitigation potential of microbial biostimulants (MBs), including arbuscular mycorrhizal fungi (AMF) and *Pseudomonas fluorescens* So_08 (PGPR), over a 52-day period using multi-omics approaches. Root exudate profiling revealed distinct metabolic changes under HM stress, which compromised soil–plant interactions. Cd stress reduced the secretion of phenylpropanoids (sum LogFC: −45.18), lipids (sum LogFC: −27.67), and isoprenoids (sum LogFC: −11–67), key metabolites in antioxidative defense, while also suppressing rhizosphere fungal populations. Conversely, Zn stress enhanced lipid exudation (such as sphingolipids and sterols, as sum LogFC of 8.72 and 9.99, respectively) to maintain membrane integrity and reshaped rhizobacterial communities. The MBs application mitigated HM-induced stress by enhancing specialized metabolite syntheses, including cinnamic acids, terpenoids, and flavonoids, which promoted crop resilience. MBs also reshaped microbial diversity, fostering beneficial species like *Portibacter* spp., *Alkalitalea saponilacus* under Cd stress, and stimulating rhizobacteria like *Aggregatilinea* spp. under Zn stress. Specifically, under Cd stress, bacterial diversity remained relatively stable, suggesting their resilience to Cd. However, fungal communities exhibited greater sensitivity, with a decline in diversity in Cd-treated soils and partial recovery when MBs were applied. Conversely, Zn stress caused decline in bacterial α-diversity, while fungal diversity was maintained, indicating that Zn acts as an ecological filter that suppresses sensitive bacterial taxa and favors Zn-tolerant fungal species. Multi-omics data integration combined with network analysis highlighted key features associated with improved nutrient availability and reduced HM toxicity under MB treatments, including metabolites and microbial taxa linked to sulfur cycling, nitrogen metabolism, and iron reduction pathways. These findings demonstrate that MBs can modulate plant metabolic responses and restore rhizosphere microbial communities under Cd and Zn stress, with PGPR showing broader metabolomic recovery effects and AMF influencing specific metabolite pathways. This study provides new insights into plant–microbe interactions in HM-contaminated environments, supporting the potential application of biostimulants for sustainable soil remediation and plant health improvement.

## 1. Introduction

In recent years, the investigation of plant–microbe interactions under environmental stresses has gained significant scientific interest, particularly in the context of HM contamination [1]. These interactions typically occur in the rhizosphere, where the biochemical processes driven by the modulation of root exudates can affect microbial activity and directly influence the plant’s ability to cope with environmental stresses such as HMs [1,2].

HMs are naturally present in soil, and some play critical roles as cofactors in numerous biological processes in both macro- and microorganisms [3]. However, excessive concentrations of HMs—primarily introduced through anthropogenic activities such as the extensive use of chemical fertilizers, livestock manures, and sludge wastewater, which are conventionally employed in agriculture to obtain fertilized soils for crop production—pose significant environmental and agricultural challenges [3]. Beyond a critical threshold, HMs act as environmental pollutants by accumulating and disrupting soil health. In agricultural systems, HM accumulation adversely affects plant metabolism, impairing critical processes such as photosynthesis, nutrient uptake, and oxidative stress mitigation. This ultimately reduces crop growth and productivity [4]. In addition, transferring HMs from moderately contaminated soils into edible plant parts introduces potential risks to human health. Moreover, HMs with high solubility and mobility may leach into groundwater, contaminating drinking water supplies and impacting aquatic ecosystems [4,5].

Several environmentally friendly strategies have been proposed to remediate HM-polluted areas, including integrated chemical–biological remediation, plant–microbe-based phytoremediation, and the application of organic biostimulants [6]. These approaches share a common goal: to reduce HM bioavailability through immobilization, chelation, and sequestration, thereby limiting their uptake by plants and other organisms [7]. Although HMs are not degraded during these processes, their conversion into less bioavailable forms mitigates their toxicity and environmental impact [7,8]. More recently, the investigation of microbial biostimulants (MBs) has gained significant attention in overcoming abiotic stress, including HM stress. The associations between plants and beneficial microorganisms, such as Plant Growth Promoting Rhizobacteria (PGPR) and Arbuscular Mycorrhizal Fungi (AMF), have demonstrated the potential to enhance plant fitness and to confer a wide range of benefits, including improved nutrient uptake, enhanced tolerance to drought and salinity stress, and effective HM remediation [2,9]. Microbial survival in HM-contaminated soils depends on specialized adaptive mechanisms that allow soil-dwelling microorganisms to thrive in high-metal environments [10]. These mechanisms involve the production of metallophores, antioxidant compounds, and stress protectants that enhance microbial survival and protect plant cellular integrity by mitigating metal toxicity. In addition, they can stimulate the synthesis of plant molecules involved in signaling pathways—such as quorum-sensing molecules and stress-related hormones—that regulate plant responses and adaptation to environmental stress [11]. Understanding these molecular signaling pathways is essential for optimizing the use of MBs in agriculture and achieving more sustainable and productive agricultural practices.

This study investigates two key objectives to address the critical knowledge gap in understanding the intricate relationship between microorganisms and plants under HM-contaminated environments: (1) to evaluate whether AMF and a metal-tolerant PGPR strain, *Pseudomonas fluorescens* So_08, can alleviate Cd and Zn stress in tomato plants, and (2) to explore the hypothesis that plants exposed to metal stress may modulate their root exudation patterns to foster symbiotic interactions with microorganisms, thereby enhancing their overall stress tolerance. Our hypothesis that stressed plants release specific metabolites to attract beneficial microbes is grounded in emerging evidence that plants adjust their root exudate profiles in response to environmental stressors [12]. By addressing these objectives, this research combines multi-omics approaches (e.g., root exudate metabolic profile and amplicon metabarcoding of rhizosphere microbial population) to elucidate how MBs mitigate metal-induced stress, with implications for sustainable agriculture and ecosystem restoration. Understanding these mechanisms advances microbial ecology and provides insights into developing microbe-based strategies to improve plant resilience in HM-contaminated soils.

## 2. Results

### 2.1. MBs Specifically Modulate the Root Exudates Profile Under Cd- and Zn-Induced Stress

Root exudates were profiled using a UHPL-QTOF-MS, leading to the annotation of more than 1200 metabolites. The list of identified compounds, including their ontology, abundances, and MS1 isotopic and MS2 spectra (where confirmed), is provided in Table S1. The dataset was inspected for overall similarities and dissimilarities among treatments using HCA (Figure S1), reporting specific effects of MB applications and their interaction with HM stress on root-exudated metabolites. Notably, two primary clusters emerged from the HCA: the first highlighted the distinct effects of PGPR application, both with and without HM exposure. In contrast, the second cluster comprised two subclusters: one representing control and Zn-exposed plants, and the other including AMF, Cd, and Cd+AMF treatments. Afterward, AMOPLS-DA was performed to assess the variance associated with the two factors (Table 1), confirming the HCA output and showing that the MB factor statistically influenced the root exudates profile, reporting 23.2% of RSS values (*p*-value = 0.01). The discrimination among MBs was made by components 1 and 4 (Tp1 and Tp4), as reported in Table 1 and Figure S1B. Furthermore, the interaction between MB and HM factors resulted as a secondary factor, with 14.4% of the RSS value capable of explaining the root exudates variation using components 2 and 6 (Tp2 and Tp6). Finally, the HM factor contributed less to the root exudate metabolites, reporting 9% of the RSS value. Interestingly, the supervised AMOPLS model highlighted 53.3% of metabolomic differences that our factors could not explain (Table 1).

Based on both supervised and unsupervised models, consolidating the less important HM factor, we decided to proceed with further analysis by separating Cd and Zn stress to strengthen our knowledge of the potential mitigation effect of both PGPR and AMF applications under single metal stress conditions. Specifically, HCA was conducted to gain general insight into MB treatments of root exudates under Cd- and Zn-induced stresses (Figure S2A,B). Accordingly, for both metal stresses, the effect of MBs was reported to have a higher implication in the exudate profile than metal stress alone, confirming the specific modulation of the root exudate profile deriving from the interaction between plant and MBs.

**Figure 1:**
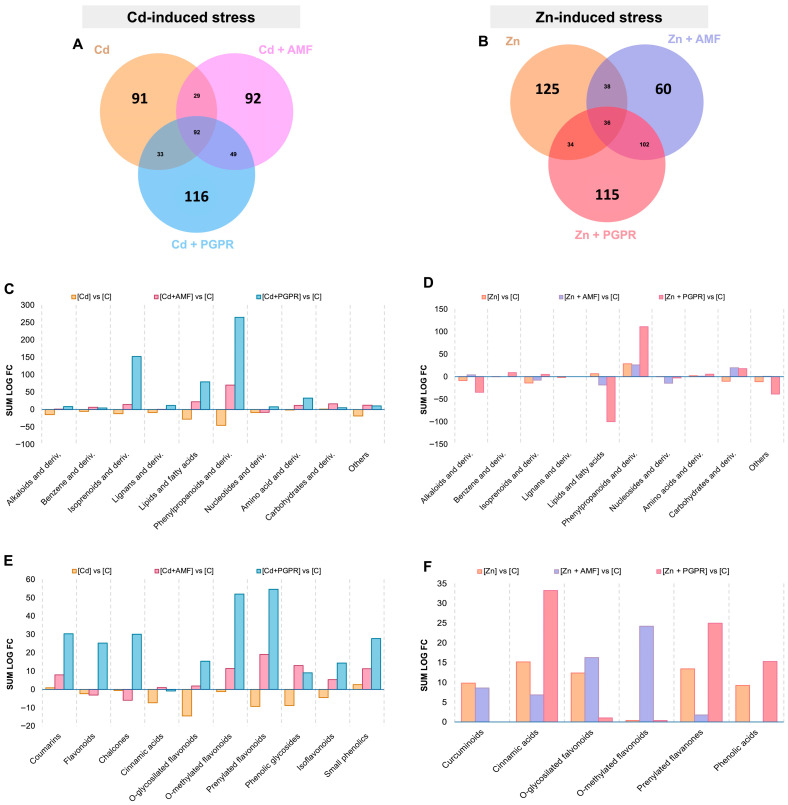
Venn diagram of variable important in projection biomarkers derived from the OPLS-DA models of (**A**) Cd-induced stress (Cd vs. C, Cd+AMF vs. C, and Cd+PGPR vs. C) and (**B**) Zn-induced stress (Zn vs. C, Zn+AMF vs. C, and Zn+PGPR vs. C) of tomato root exudates. The unique compounds were classified in their compound ontologies and represented in bar plots for the (**C**,**D**) overall discriminant exudates profile and with a focus on (**E**,**F**) phenylpropanoids, as Cd- and Zn-induced stress, respectively. Abbreviation: AMF = Arbuscular Mycorrhizae Fungi, PGPR = Plant Growth Promoting Rhizobacteria, C = Control.

The root exudate profile of tomato plants exposed to Cd and Zn stress was analyzed using the Orthogonal Partial Least Squares–Discriminant Analysis (OPLS-DA) model to identify the most discriminant biomarkers associated with the single treatment, focusing on pairwise comparisons between Cd or Zn, Cd+AMF or Zn+AMF, and Cd+PGPR or Zn+PGPR treatments versus the control (Figure S3). The model’s performance was evaluated based on goodness-of-fit (R^2^Y) and predictive ability (Q^2^Y) for each OPLS-DA model. Then, the OPLS-DA models were statistically validated using cross-validation ANOVA, as summarized in Table S2 for Cd and Table S3 for Zn-induced stress. This study specifically identified VIP makers across the pairwise comparative conditions (VIP score > 1.2), representing metabolites that contributed the most to discrimination for each factor. These metabolites have been selected, and a Venn diagram was then used to exclude the contribution of the control effect, allowing for the identification of unique metabolites, as reported in Figure 1 for Cd stress Figure 1A and for Zn stress Figure 1B. Furthermore, these unique metabolites were integrated with log FC values and classified into metabolite classes to provide deeper insights into the effect of MB treatments.

As reported in Figure 1C, the Cd-exposed plant exhibited reduced secretion levels of phenylpropanoids, primarily represented by flavonoids, isoflavonoids, and cinnamic acids (Figure 1E). This reduction extended to other metabolite classes, including lipids, fatty acids, isoprenoids, alkaloids, amino acids, and other secondary compounds. Specifically, within the isoprenoid category, monoterpenoids and tetraterpenoids were particularly affected by Cd stress (Figure S4A). Moreover, lipid secretion was broadly suppressed under Cd stress, including fatty acids, steroids, phytohormones (indole-3-acetic acids; Figure S5), sphingolipids, and glycolipids (Figure S4B). Interestingly, the co-exposure of Cd stress with MBs reversed this trend, with PGPR application proving particularly effective. Specifically, under these conditions, the secretion levels of key metabolite classes shifted significantly, mirroring those observed under Cd stress but in the opposite direction compared to the control.

In the case of Zn-induced stress, the tomato plant did not report a uniform modulation of root-secreted metabolites. Instead, specific metabolic classes, including isoprenoids, fatty acids, and carbohydrates, were detected as having divergent modulation trends compared to MBs. Among them, the Zn+PGPR reported the most significant impact on the root exudates, characterized by a decrease in alkaloids and lipid secretion alongside a notable increase in phenylpropanoid and isoprenoid metabolite classes (Figure 1D). Zn+PGPR treatment enhanced the secretion of diterpenoids, triterpenoid saponins, and terpene glycosides (Figure S4C). In contrast, triterpenes were decreased under Zn+PGPR and Zn+AMF applications, suggesting that these MBs may suppress specific branches of the isoprenoid biosynthetic pathway, potentially to reallocate resources toward other stress-related metabolic processes (Figure S4C). Regarding the phenylpropanoid class, Zn+PGPR increased the release of cinnamic acids, prenylated flavonoids, and phenolic acids (Figure 1F). In contrast, Zn+AMF treatment predominantly promoted the secretion of O-methylated flavonoids. Finally, considering lipids and fatty acids, Zn+PGPR induced a decrease in long and very long-chain fatty acids, sphingolipids, and steroids (Figure S4D).

### 2.2. MBs Specifically Modulate the Rhizosphere Microbial Population Under Cd and Zn-Induced Stress

The amplicon sequencing analysis identified HMs- and MBs-induced shifts in bacterial and fungal populations in the rhizosphere. 16S samples yielded an average of 16,000 high-quality reads per sample with an average coverage of 92.8%. ITS amplicons produced an average of 42,000 high-quality reads per sample, with an average coverage of 93.0%. In both cases, the high coverage indicates that a substantial portion of the microbial diversity was captured in the analysis.

**Figure 2:**
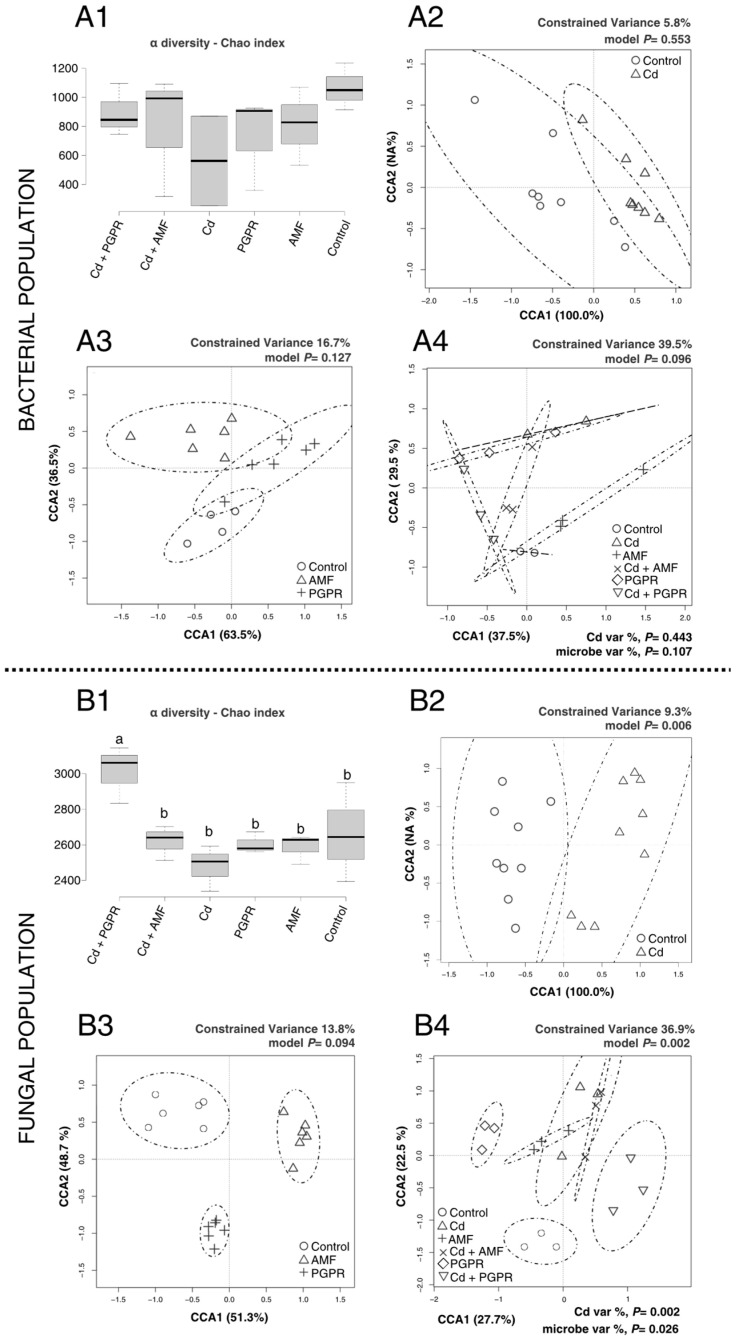
Effects of Cd stress and MBs on rhizosphere microbial diversity in tomato plants. Panels (**A1**–**A4**) correspond to bacterial community analyses, while (**B1**–**B4**) correspond to fungal community analyses. (**A1**) Bacterial α-diversity (Chao index). Different lowercase letters above boxplots indicate statistically significant differences between treatments (*p* < 0.05). (**A2**) Bacterial β-diversity (Cd vs. control). (**A3**) Bacterial β-diversity among microbial treatments (PGPR, AMF, and control). (**A4**) Comprehensive bacterial β-diversity considering all treatments. (**B1**) Fungal α-diversity analysis (Chao index), with different lowercase letters denoting significant differences (*p* < 0.05). (**B2**) Fungal β-diversity (Cd vs. control). (**B3**) Fungal β-diversity among microbial treatments (PGPR, AMF, and control). (**B4**) Comprehensive fungal β-diversity analysis.

**Figure 3:**
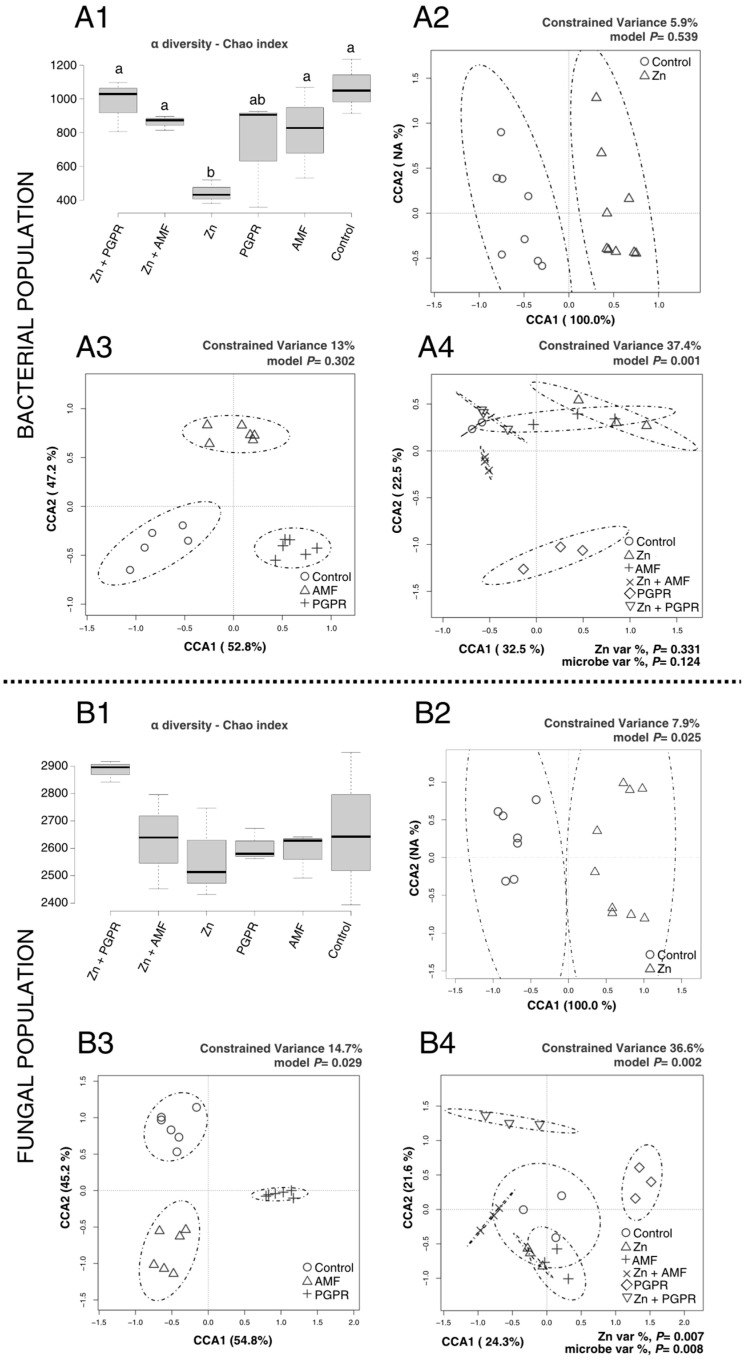
Effects of Zn stress and MBs on rhizosphere microbial diversity in tomato plants. Panels (**A1**–**A4**) correspond to bacterial community analyses, while (**B1**–**B4**) correspond to fungal community analyses. (**A1**) Bacterial α-diversity (Chao index). Different lowercase letters above boxplots indicate statistically significant differences between treatments (*p* < 0.05). (**A2**) Bacterial β-diversity (Zn vs. control). (**A3**) Bacterial β-diversity among microbial treatments (PGPR, AMF, and control). (**A4**) Comprehensive bacterial β-diversity considering all treatments. (**B1**) Fungal α-diversity (Chao index), with different lowercase letters denoting significant differences (*p* < 0.05). (**B2**) Fungal β-diversity (Zn vs. control). (**B3**) Fungal β-diversity among microbial treatments (PGPR, AMF, and control). (**B4**) Comprehensive fungal β-diversity analysis.

The results showing microbial community shifts in tomato rhizosphere soils treated with Cd and Zn are illustrated in Figure 2. The Cd contamination did not significantly impact bacterial α-diversity (Figure 2(A1)), indicating that the abundance and richness of bacterial populations were not affected. The same result was reported for β-diversity (Figure 2(A2)). Interestingly, β-diversity analysis revealed that bacterial populations in AMF-treated soils clustered separately from those in the control (Figure 2(A3)), suggesting a partial effect of AMF on bacterial community composition. Regarding the Zn contamination, the bacterial populations were significantly reduced, as suggested by α-diversity when compared to regular soil (Figure 3(A1)). Interestingly, the application of MBs under Zn stress, especially PGPR, resulted in an overall increase in bacterial α-diversity, partially restoring diversity to levels closer to those of the control soils. This suggests that PGPR and AMF mitigated some of the negative effects of Zn contamination on bacterial populations. The β-diversity analysis provided additional insights into bacterial community composition. In Figure 3(A4), the comprehensive β-diversity analysis revealed significant differences (*p* < 0.01) among treatments, identifying four groups: (1) PGPR, (2) Zn+AMF, (3) a group comprising Control, Zn+PGPR, and AMF, and (4) a group comprising AMF+Zn. These results suggest that Zn contamination influences bacterial populations, with microbial treatments, particularly PGPR, helping to mitigate the effects of Zn on bacterial diversity and community composition.

For fungal populations, the response to Cd treatments was more pronounced. The α-diversity analysis (Figure 2(B1)) revealed that Cd+PGPR-treated soils had significantly higher fungal diversity compared to other treatments (*p* < 0.01). The β-diversity analysis demonstrated that Cd significantly influenced fungal community composition (Figure 2(B2)), with distinct clustering between fungal populations in Cd-treated soils and the control (*p* < 0.01). While microbial treatments also produced distinct clusters (Figure 2(B3)), even though these differences were not statistically significant. The comprehensive β-diversity analysis (Figure 2(B4)) revealed significant clustering (*p* < 0.01), identifying four cluster groups: (1) Control, (2) PGPR, (3) Cd+PGPR, and (4) a cluster comprising Cd, Cd+AMF, and AMF. These results indicate that the amount of Cd added was insufficient to cause a significant reduction in microbial communities in the tomato rhizosphere, although a trend of reduced diversity was observed. Adding PGPR in Cd-contaminated soils significantly increased fungal α-diversity compared to other treatments.

In the case of Zn-induced stress, the fungal populations were not affected by the treatments, as revealed by the α-diversity (Figure 3(B1)). However, β-diversity analysis revealed significant shifts in fungal community composition in response to Zn. In Figure 3(B2), fungal populations in Zn-treated soils clustered significantly differently from the control (*p* < 0.05). Microbial treatments also significantly influenced fungal β-diversity (Figure 3(B3)), with distinct clustering observed between fungal populations treated with PGPR, AMF, and control (*p* < 0.05). The comprehensive β-diversity analysis (Figure 3(B4)) revealed three distinct clusters: (1) Zn+PGPR, (2) PGPR, and (3) a larger cluster comprising the remaining treatments. These results indicate that Zn contamination induced significant shifts in fungal community composition, although α-diversity was unaffected. Microbial treatments, particularly PGPR, appeared to influence fungal populations despite the toxic effects of Zn.

### 2.3. Multi-Omics Data Integration of Fungal, Bacterial, and Exudates

**Figure 4:**
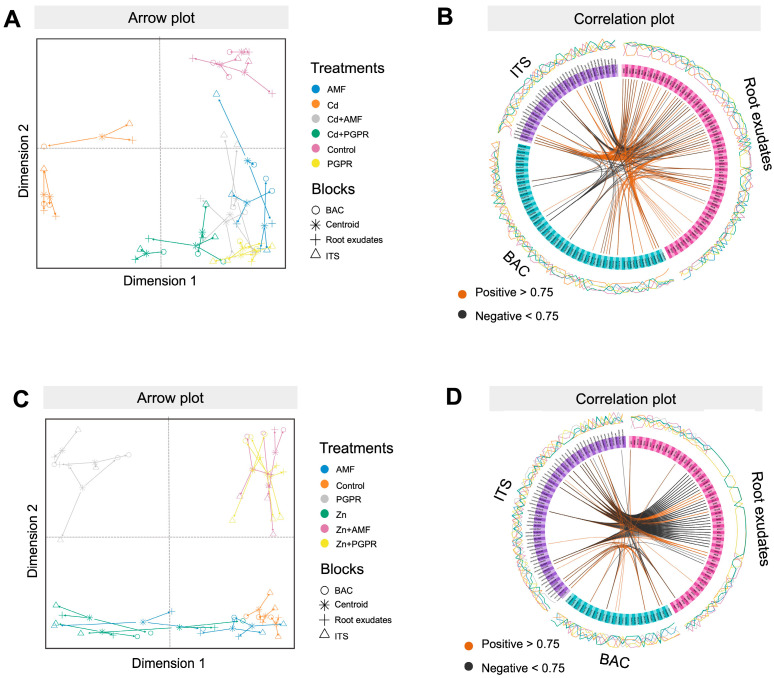
DIABLO-based data integration models for the exudate profile, rhizosphere bacterial, and fungal metagenomics datasets of the tomato plant affected by Cd and Zn stress. Arrow plot of the three integrated datasets discriminating among different microbial biostimulants (AMF and PGPR) in tomato plants grown under Cd (**A**) and Zn (**C**) stress, respectively. Circos correlation plot of the three datasets, where the selected features are involved in positive and negative correlations, for Cd (**B**) and Zn (**D**) stress. Abbreviation: AMF = Arbuscular Mycorrhizae Fungi, PGPR = Plant Growth Promoting Rhizobacteria, C = Control.

To find a direct correlation between metabolomics and metagenomics datasets, a horizontal data integration approach based on the DIABLO data integration framework was employed. This workflow was applied separately for both Cd- and Zn-induced stress (Figure 4) to determine the influence of MBs on the root exudate profile and fungal and bacterial soil rhizosphere populations of tomato plants.

The DIABLO model for Cd demonstrated high correlation scores, averaging 0.89 and 0.84, across the first two components. These high correlations emphasize the robustness and reliability of the integrated omics model in distinguishing between treatments. The arrow plot highlighted a substantial contribution from all three datasets, as indicated by the minimal arrow distances between the omics data (Figure 4A). Specifically, the first component played a central role in discriminating Cd stress from other conditions, while the second component was attributed to the discrimination between MB-treated samples and the control (Figure 4A). Furthermore, the joint circos correlation analysis revealed notable positive and negative correlations among features (r > |0.75|; Figure 4B), underscoring the interconnectivity of metabolic pathways and the biological signatures shared in response to treatments. The features that are significantly responsible for discriminating between different treatments are reported in Figure S6A, and a complete list of loading features is provided in Table S4.

The DIABLO model for Zn exhibited strong correlations, with average scores of 0.87 and 0.93 for the first two components. The first component significantly distinguished PGPR treatments, which clustered separately from other treatment groups, including Zn+AMF and Zn+PGPR plants. Meanwhile, the second component exhibited great discrimination potential for the control and MB samples, thereby highlighting distinct metabolic and microbial profiles across these conditions (Figure 4C). The complete list of features for all three datasets and components is provided in Table S5. The circos correlation analysis revealed a complex network of positive and negative correlations among features across the three datasets (r > |0.75|; Figure 4D). The loading plots (Figure S6B) identified significant features driving treatment discrimination, highlighting specific correlated features that were particularly significant under PGPR, MB, and combined Zn-stress conditions (Table S5).

### 2.4. Network Analysis of Highly Discriminant Features Obtained from Multi-Omics Data Integration in Cd- and Zn-Stressed Models

**Figure 5:**
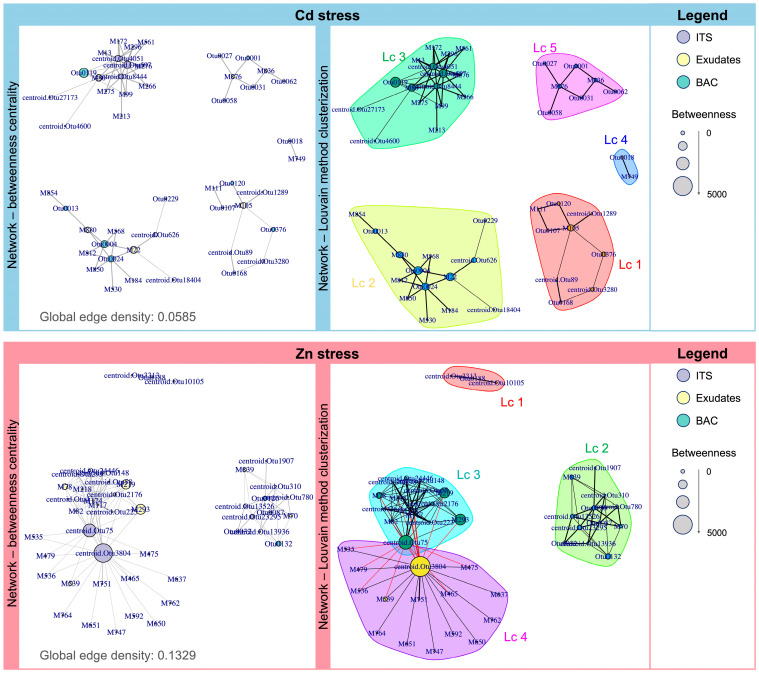
Network analysis, represented by betweenness centrality and the Louvain clusterization method, for Cd- and Zn-induced stress. Nodes represent root exudate metabolites (yellow), bacterial taxa (turquoise), and fungal taxa (purple). Edges represent significant correlations (positive or negative) between nodes. To enhance interpretability, node size was scaled based on the degree of betweenness of each feature, and edge thickness reflected the strength of the correlations.

The highly correlated features (r > |0.75|) derived from the DIABLO model were finally subjected to network analysis to visualize the intricate relationships between root exudate metabolites and the rhizosphere microbial community, including bacterial and fungal populations. This integrative approach enabled us to identify key associations within the root–soil–microbe system under HM stress, and when combined with MB treatments (Figure 5). Network topology metrics, such as global edge density and betweenness centrality, were calculated to identify hubs, key metabolites, and microbial species that play central roles in the network structure. Finally, the network was subjected to the Louvain clusterization methodology to detect communities within the network.

Regarding Cd-derived stress, the resulting network exhibited a global edge density of 5.85%, reflecting sparse but meaningful connectivity among metabolites and microbial taxa. Notably, *Cedecea davisae* was identified as the hub of betweenness centrality in the network, underlining its important role in mediating interactions between key nodes. To further explore the network structure, the Louvain clustering method was applied, revealing five distinct communities characterized by highly interconnected metabolites and microbial species. The complete list of features belonging to each cluster is reported in Table S6. In the first cluster (Lc1), significant associations were observed between specific metabolites, such as linoleic acid and indoles and derivatives, and bacterial species, including *Capillibacterium thermochitinicola* and *Sphaerobacter* spp. Cluster 2 (Lc2) further emphasizes the central roles of *Portibacter* spp. and *Flavitalea gansuensis* in response to Cd-induced stress, exhibiting significant connections with diverse metabolites, including xanthophylls, terpenoids, cinnamic acids, and flavonoids. The third cluster (Lc3) revealed a complex interplay between root exudates, fungal populations, and bacterial taxa under MBs and HM treatments. This cluster highlighted the importance of *Aspergillus nidulans* and *Scedosporium apiospermum*, which exhibited strong associations with *Cedecea davisae* and specific root exudates, including flavonoid *O*-glycosides, steroids, saponins, and coumarins. These findings underscore the synergistic roles of fungal and bacterial species in metabolite interactions and plant stress responses. Finally, clusters 4 and 5 (Lc4 and Lc5) were associated with bacterial communities dominated by *Desulfovulcanus* spp., *Geobacillus thermodenitrificans*, and *Acidibacter ferrireducens*, taxa that are likely involved in sulfur cycling, nitrogen metabolism, and iron reduction processes under Cd stress.

Concerning Zn-driving stress (Figure 5), the resulting network exhibited a global edge density of 13.29%, reflecting denser connectivity than Cd stress. *Entoloma sororpratulense* was identified as the betweenness centrality hub, highlighting its role in mediating interactions between plant and microbial communication under Zn stress conditions. The Louvain clustering revealed four distinct communities. The detailed list of features associated with each cluster is provided in Table S7. Among these clusters, the second cluster (Lc2) reported a prominent association between fungal taxa, including *Microstoma longipilum* and *Phylloporus castanopsidis*, and rhizobacterial species such as *Phototrophicus methaneseepsis* and *Nitratireductor aestuarii*. These microbial taxa demonstrated significant connections with xanthones and prenylated flavanones. Notably, the third and fourth clusters (Lc3 and Lc4) were represented by two highly connected fungal taxa, *Microdochium musae* and *Entoloma sororpratulense*, which have a high degree of connectivity. These are positively correlated with specific metabolites, including amino acids and derivatives, cucurbitacin glycosides, and sesquiterpenoids. Conversely, these taxa exhibited negative correlations with long-chain fatty acids, triacylglycerols, tocopherols, triterpenoids, and ergosterols.

## 3. Discussion

The MBs applied in this study effectively enhanced plant growth and stress tolerance through diverse biochemical and physiological mechanisms, as previously demonstrated [13]. However, the specific mechanism underlying the MB–plant–soil remains insufficiently explored. In this study, the combined use of *P. fluorescens* and AMF product was evaluated in tomato plants exposed to Cd and Zn contamination, with emphasis on how MBs reshape root exudation patterns to promote symbiotic interactions and strengthen stress resilience. Importantly, AMOPLS variance partitioning revealed that MBs accounted for a greater proportion of exudate variation than the metal factor itself, highlighting that microbial partners can strongly reprogram root chemical composition even under heavy metal (HM) stress. This finding supports the concept of stress-induced recruitment, whereby plants modify their metabolic profiles to attract beneficial microorganisms capable of mitigating environmental constraints [12].

### 3.1. Plant–Microbial Communication Mechanism Under Cd and Zn Stress

The responses of tomato plants to Cd and Zn contamination revealed distinct metabolic and ecological strategies, confirming that the identity of heavy metals determines the mode of plant–microbe interaction. The two metals differ fundamentally in their biochemical behavior. Cd, being non-essential and highly reactive, disrupts enzymatic cofactors and displaces essential ions [14], whereas Zn, though essential, perturbs cellular homeostasis only at supra-physiological concentrations [15]. Therefore, Cd imposed a global inhibition of specialized metabolism and signaling, while Zn induced a targeted remodeling of specific metabolic and microbial processes [14,15,16]. The comparison between these two metals indicates how variations in essentiality and redox behavior shape the plant’s biochemical allocation, its capacity for rhizosphere signaling, and the structure of associated microbial communities.

Root exudates profiling showed that Cd stress reduced the secretion of phenylpropanoids, lipids, and isoprenoids. These metabolite classes are fundamental for antioxidative defense, cell wall reinforcement, and stress signaling [17]. Such coordinated suppression suggests that Cd interferes with NADPH availability and carbon partitioning, diverting primary metabolism toward repair and detoxification rather than secondary metabolite synthesis [18]. Their reduction points to a reallocation of carbon and energy resources from secondary metabolism toward basic cellular maintenance. The decline in phenolic acids, flavonoids, and cinnamate derivatives reflects a weakened antioxidant capacity and reduced ability to detoxify reactive oxygen species (ROS) [17,19]. Likewise, the decrease in sphingolipids, sterols, and fatty acids suggests impaired membrane integrity and lipid signaling, limiting the plant’s ability to perceive and respond to external cues [20,21]. Given that sphingolipids also act as bioactive messengers in programmed cell death and pathogen defense, their reduction implies a broader disruption of cell signaling cascades beyond structural effects [22]. The down-accumulation of isoprenoid-derived carotenoids and xanthophylls, accessory isoprenoids involved in light harvesting [23], further indicates that Cd stress redirects metabolic fluxes toward maintaining photosynthetic stability rather than producing signaling terpenoids. These combined changes are consistent with previous findings that Cd suppresses enzymes of the phenylpropanoid pathway, disturbs lipid homeostasis, and inhibits isoprenoid synthesis [17,21,24]. Cd therefore restricts the pool of exuded antioxidants and signaling molecules, attenuating the chemical communication that coordinates the root–microbe interface.

In contrast, Zn exposure did not result in a general suppression of secondary metabolism but rather in a selective reorganization of metabolic priorities. While certain isoprenoid and carbohydrate pathways were reduced, phenylpropanoids, sphingolipids, and sterols were significantly increased. This divergent pattern indicates that, under Zn stress, plants actively redirect carbon fluxes toward antioxidant phenolics and membrane-reinforcing lipids as part of a compensatory response. Such a shift likely serves to preserve membrane stability, maintain signaling competence, and control oxidative stress [24,25]. The increased synthesis of structural lipids and antioxidant phenolics suggests that plants exposed to Zn stress undergo metabolic adjustment rather than systemic inhibition, in which biosynthetic resources are reallocated from growth-related to defense-related pathways. Zn toxicity thus induces a controlled remodeling of metabolism characterized by enhanced production of defense-related metabolites and lipids, consistent with the metal’s moderate stress intensity and its partial physiological role as an enzymatic cofactor [25,26].

The modifications in root exudation were reflected in the composition and diversity of rhizosphere microbial communities. Under Cd stress, bacterial diversity remained relatively stable, confirming their resilience to Cd. However, fungal communities exhibited greater sensitivity, with a decline in diversity in Cd-treated soils and partial recovery when microbial biostimulants were applied. This disproportionate fungal response aligns with the greater dependency of fungal guilds on plant-derived aromatic compounds and root-secreted carbohydrates as carbon and signaling sources. A reduction in such exudates weakens their colonization potential, leading to simplified fungal communities [27]. Conversely, Zn stress caused a significant decline in bacterial α-diversity, while fungal diversity was maintained but accompanied by compositional shifts, indicating that Zn acts as an ecological filter that suppresses sensitive bacterial taxa and favors Zn-tolerant fungal species [28]. The contrasting sensitivity of microbial groups under Cd and Zn is thus rooted in their physiological traits. Bacteria are more vulnerable to ionic interference with metalloproteins and oxidative enzymes, whereas fungi can tolerate moderate Zn exposure due to cell wall binding and polyphosphate sequestration [29]. These results demonstrate that Cd primarily affects the plant metabolic contribution to microbial signaling, while Zn alters microbial population dynamics more directly, leading to distinctive community restructuring in each case.

The integration of metabolomic and metagenomic datasets through the DIABLO framework provided a mechanistic overview of how these chemical and microbial components interact. The strong correlations among exudates, bacteria, and fungi (r = 0.87–0.94) indicate a high degree of coordination between plant metabolic adjustments and microbial responses. Under Cd stress, the network topology was sparse but highly organized, dominated by bacterial hubs such as *Cedecea davisae*, which connected coumarins, flavonoid glycosides, and diterpenoids with microbial partners including *Aspergillus nidulans* and *Scedosporium apiospermum*. These associations may suggest a restructured communication framework in which a limited set of metabolites mediates interactions between plants and specific microbial taxa involved in sulfur, nitrogen, and iron cycling [30,31,32]. Such restructuring implies that, under Cd limitation of exudate diversity, the rhizosphere relies on “core interaction modules” centered around nutrient-transforming microbes that compensate for the plant’s restricted metabolic flexibility. These modules likely enhance redox buffering capacity and maintain trace element bioavailability [33].

Under Zn stress, the network exhibited higher connectivity (edge density = 13.29%), with a predominance of fungal nodes such as *Entoloma sororpratulense* and *Microdochium musae*. These fungi correlated positively with amino acids, cucurbitacin glycosides, and sesquiterpenoids but negatively with long-chain fatty acids, tocopherols, and sterols, indicating a redistribution of carbon fluxes from storage lipids toward secondary metabolites with protective and signaling functions. The involvement of bacterial taxa such as *Phototrophicus methaneseepsis* and *Nitratireductor aestuarii* within these networks further demonstrates how Zn stress reorganizes cross-kingdom interactions around nutrient turnover and oxidative regulation [34,35].

These findings indicate that Cd stress gives rise to a bacterially dominated, resource-limited network primarily oriented toward redox buffering, whereas Zn stress fosters a fungal-centered, metabolically enriched network emphasizing nutrient exchange and antioxidative maintenance. This contrast provides a mechanistic basis for understanding how microbial biostimulants modulate and reconfigure these networks under metal-induced stress.

### 3.2. Mitigation Mechanisms of Microbial Biostimulants

The application of MBs, *P. fluorescens*, and AMF, substantially mitigated the adverse effects of both metals by re-establishing plant–microbe communication and restoring metabolic balance. Although both microorganisms improved plant tolerance, their mechanisms of action differed in scope and specificity. PGPR primarily acted as a broad metabolic activator, enhancing the quantity and diversity of root exudates, while AMF functioned as a selective modulator, refining key biochemical pathways related to signaling and membrane integrity.

In Cd-contaminated soils, PGPR inoculation partially reversed Cd-induced metabolic inhibition by reactivating the synthesis of phenolic acids, flavonoids, coumarins, and various terpenoid derivatives. These metabolites have dual defensive and ecological functions, acting as potent antioxidants, Cd-chelating agents, and chemical attractants for beneficial microorganisms [25,27,28]. Enhanced secretion of glycosylated terpenes, which are more soluble and stable in the soil matrix, extends the persistence of signaling molecules and facilitates long-distance microbe recognition and metal immobilization [28]. PGPR also stimulated the exudation of gibberellin- and serotonin-like metabolites associated with phytohormonal regulation of growth and stress adaptation [36,37]. These effects indicate that PGPR improves both the plant’s chemical defense capacity and its ability to recruit beneficial microorganisms. At the microbial community level, this biochemical recovery coincided with higher fungal α-diversity and the re-emergence of bacterial network hubs associated with sulfur and iron cycling. The coupling between phenolic-rich exudates and microbial functional groups suggests that PGPR not only counteracts Cd toxicity but also facilitates the re-establishment of ecological feedback loops that stabilize the rhizosphere [30,31,32].

AMF inoculation under Cd stress induced a more targeted biochemical signature dominated by cinnamic acids, phenolic glycosides, and sphingolipids, metabolites crucial for stress perception and cell signaling [19,26]. Cinnamic acid derivatives serve as precursors for lignin and flavonoid biosynthesis and have been shown to promote AMF colonization and arbuscule formation [38,39]. Their accumulation thus reflects a positive feedback loop where plant-derived metabolites enhance fungal symbiosis, which in turn improves nutrient uptake and detoxification. The increase in sphingolipids indicates active remodeling of plasma membranes that improves their stability and communication capacity under metal stress [21,24]. AMF therefore contributes primarily to qualitative improvement—enhancing membrane function, vesicle trafficking, and signaling fidelity rather than increasing the overall exudate output. This specialized modulation complements the PGPR-induced quantitative stimulation, demonstrating a division of labor between microbial partners in rebuilding both the biochemical and physical infrastructure of the rhizosphere.

Under Zn stress, PGPR again promoted secondary metabolism, especially the production of phenolic acids, prenylated flavanones, and xanthones, while down-accumulating long-chain fatty acids and steroids. This adjustment rebalanced carbon allocation from structural lipids toward antioxidants and metal-binding compounds, strengthening the plant’s capacity to manage Zn excess [40,41]. The resulting enrichment in phenolics and xanthones enhances both radical-scavenging and Zn-binding capacity, reducing the pool of free ionic Zn in the rhizosphere and improving redox stability. In parallel, PGPR-mediated metabolic restoration partially recovered bacterial α-diversity, consistent with the re-establishment of nutrient-cycling functions and reduced oxidative load. The DIABLO integration revealed strong correlations between PGPR-linked metabolites and bacterial taxa such as *Phototrophicus methaneseepsis* and *Hydrogenophaga temperata*, confirming the reactivation of carbon and nitrogen turnover pathways crucial for microbial cooperation and soil resilience [32,34]. AMF, in turn, enhanced O-methylated and O-glycosylated flavonoids, compounds with higher stability and antioxidative potency [42]. These modifications optimize the phenylpropanoid pool for sustained antioxidant activity and long-distance signaling in Zn-rich soils. AMF also contributed to fungal network coherence, supporting highly connected taxa such as *Entoloma* and *Microdochium* that anchor cross-kingdom interactions. Together, PGPR and AMF reorganized the rhizosphere from a stress-driven to a metabolically coordinated system.

The two microbial biostimulants act through complementary but convergent pathways. PGPR exerts a broad-spectrum stimulatory effect, reactivating multiple metabolic pathways and enhancing the availability of substrates and signals that support diverse microbial populations. AMF exerts a precision effect, targeting membrane composition and key signaling molecules to stabilize communication and symbiotic performance. Both converge on the reinforcement of phenylpropanoid and terpenoid metabolism, but PGPR drives quantitative expansion, whereas AMF ensures biochemical selectivity and signal integrity. This complementary relationship explains why co-inoculation frequently yields additive or synergistic improvements in plant growth and stress tolerance [9,13,43]. From a systems perspective, PGPR and AMF together reconfigure the plant–microbe interface into a more connected and resilient network. PGPR reopens previously suppressed metabolic routes, increasing the chemical diversity available for microbial colonization and metal immobilization. AMF strengthens the physical and signaling integrity of membranes, ensuring that the increased metabolic activity translates into stable inter-kingdom cooperation. The outcome is a rhizosphere characterized by higher diversity, functional redundancy, and efficient nutrient cycling, as evidenced by the enrichment of microbial taxa involved in sulfur, nitrogen, and iron transformations [12,27,44]. This reorganized structure not only mitigates Cd and Zn toxicity but also enhances plant nutrient acquisition and soil biochemical resilience.

Despite the promising outcomes, several unanswered questions remain regarding the long-term stability of MB-induced microbial shifts, the specificity of metabolite–microbe interactions under different HM concentrations, and the scalability of these findings across different crop species and soil types. Future research should explore the temporal dynamics of root exudation, the resilience of microbial consortia under field conditions, and the integration of MBs into holistic phytoremediation strategies tailored to diverse agroecosystems.

## 4. Materials and Methods

### 4.1. Plant Growth Condition

Tomato plants (*Solanum lycopersicum* L., cv. Heinz 3402) were grown for 52 days under natural open field conditions at the experimental station of Università Cattolica del Sacro Cuore (Piacenza, Italy), as detailly reported in our previous work [13]. Briefly, the setup considered two variables: the stress level induced by HMs, which included untreated soil, soil contaminated with zinc (Zn), and soil contaminated with cadmium (Cd); and the microbial treatment, comprising untreated plants, plants inoculated with a PGPR, and plants inoculated with AMF. The experiments were organized in a completely randomized design, with three biological replicates for each experimental unit, resulting in a total of 27 pots.

MBs were applied by irrigation during transplanting, specifically AMF treatment involved a commercial product sourced from Athens, specifically Agrotecnologia Naturales SL (Tarragona, Spain). This product is formulated using *Rhizoglomus irregulare* BEG72 and *Funneliformis mosseae* BEG234 (Aegis Sym irriga^®^, Tarragona, Spain), and includes 700 spores per gram for each species. The formulations were applied according to the label instructions, entailing a single application of 0.1 g per plant. While the PGPR-based treatment was a *Pseudomonas fluorescens* So_08, originally isolated from HM-polluted soil, as previously described by [45]. The bacterial strain was grown in Tryptic Soy Broth medium with orbital shaking at 180 rpm and 28 °C for 24 h. Afterward, the cells were collected, washed thrice, and re-suspended in sterile water to reach a final concentration of 10^9^ CFU mL^−1^.

Upon sowing, two weeks were considered enough for the microorganisms to establish an interaction with the plant. During the same period, pots were fertilized using a half-strength Hoagland solution once a week. The procedure for preparing the Hoagland solution is described in this work [46]. After this period, HMs were distributed weekly for five consecutive weeks until the final concentrations of 100 mg kg^−1^ for Cd and 400 mg kg^−1^ for Zn were reached, using reagents CdCl_2_ and ZnSO4 (Merck KGaA, Darmstadt, Germany), following the methodology outlined by Alengebawy et al. (2021) [47].

### 4.2. Exudate Profiling

Root exudates were collected after washing the roots using tap water and rinsing them with distilled water to remove any traces of soil. They were then transferred to 250 mL beakers containing 100 mL of distilled water and left exudating for 4 h continuously, covering the beaker with aluminum foil to keep the roots in the dark. After 4 h, the root exudate solutions were centrifuged at 5000× *g* for 15 min, filtered at 0.22 μm, and freeze-dried for the following analysis. 50 mL of freeze-dried exudate solution was re-suspended into 1 mL of 50% methanol (*v*:*v*) and the exudate profiling was conducted using a 6560-drift tube-ion mobility-quadrupole-time of flight-high resolution mass spectrometer (DTIM-UHPLC-QTOF-HRMS; Agilent Technologies, Santa Clara, CA, USA) with an injection volume of 19 µL, as described in detail in the Supplementary Materials. Data annotation and MS/MS structural confirmations were performed with MS-DIAL software (version 4.90). This process involved automated peak detection (against pooled QC) and putative annotation through spectral matching with accessible databases such as BMDMS-NP, the Fiehn/Vaniya natural product library, and GNPS, as detailed in the Supplementary Materials [48].

### 4.3. Amplicon Sequencing

Rhizosphere samples were collected, isolating the soil adhering to the roots after carefully shaking off bulk soil. Total DNA was extracted from 500 mg of rhizosphere soil using the FastDNA™ SPIN Kit for Soil (MP Biomedicals, Santa Ana, CA, USA), and yields were quantified with the Quant-iT™ HS ds-DNA assay kit (Invitrogen, Waltham, MA, USA) and a QuBit™ fluorometer (Invitrogen, Waltham, MA, USA). Bacterial and fungal populations were assessed via nested PCR with barcoded universal primers targeting the V3-V4 region for bacteria and ITS1 for fungi. PCR products were pooled, purified using the SPRI method, and sequenced on an Illumina MiSeq platform (Illumina Inc., San Diego, CA, USA), generating 300 bp paired-end reads. A more detailed protocol is provided in the Supplementary Material.

### 4.4. Multi-Omics Data Integration

To integrate the datasets (blocks) derived from root exudate profile (EX) and metagenomics analyses of bacteria (BAC) and fungi (ITS), the Data Integration Analysis for Biomarker discovery using Latent variable approaches for Omics studies (DIABLO) framework was implemented within the “mixOmics” R package (version 6.22). Separate models were constructed for Cd and Zn pollutions, focusing on the six treatments that exhibited the most significant outcomes in individual analyses: control, HM, AMF, PGPR, HM+AMF, and HM+PGPR. The DIABLO model optimization is reported in the Supplementary Materials.

### 4.5. Statistical Analysis

Root exudate profile raw data were processed, transformed, and normalized using Mass Profiler Professional 12.6 (Agilent Technologies). The supervised ANOVA Multiblock Orthogonal Partial Least Squares (AMOPLS) was carried out using the rAMOPLS package in R (version 4.2.1). Subsequently, unsupervised hierarchical cluster analysis (HCA), employing Euclidean distance and Ward’s linkage method, was conducted to explore sample patterns separately for Cd- and Zn- driving stress models. The supervised orthogonal projection to latent structures discriminant analysis (OPLS-DA), using the SIMCA software (v.16, Umetrics^®^, Malmö, Sweden), were carried out separately for [HM stress vs. C], [HM+AMF vs. C], and [HM+PGPR vs. C]. Then, Variable Importance in Projection (VIP) markers, VIP score > 1.2, were selected for both Cd and Zn groups. More information is reported in the Supplementary Materials. The Venn diagram was applied to identify unique metabolites associated with each treatment: HM, HM+AMF, and HM+PGPR. Finally, VIP markers associated with each treatment were categorized into metabolite classes and visualized using bar plots that compare [HM vs. Control], [HM+AMF vs. Control], and [HM+PGPR vs. Control] for both Cd and Zn treatment groups.

Statistical evaluations of HTS data were carried out using Mothur software alongside R v3.0.02, incorporating the Vegan package [13]. Further methodological specifics are detailed in Vasileiadis et al., 2015 [49]. Alpha-diversity indices such as Shannon’s Index, Observed Richness (S), Simpson’s Diversity Index (D), and Chao’s Index were considered to examine variations in microbial communities for each treatment. Principal Component Analysis (PCA) was applied to investigate unconstrained groupings among samples, while Canonical Correspondence Analysis (CCA) was employed to evaluate the influence of different treatments on the observed diversity.

## 5. Conclusions

This study provides new insights into how MB application can influence plant metabolic responses and sustain rhizosphere microbial communities under HM stress. Tomato plants exposed to Cd and Zn significantly altered root exudate profiles, consequently influencing bacterial and fungal diversity. Applying the PGPR *Pseudomonas fluorescens* So_08 and AMF demonstrated distinct yet complementary effects in modulating plant–microbe interactions in HM-contaminated soils. Especially the PGPR treatment in both Cd- and Zn-contaminated soil broadly restored rhizosphere microbial biodiversity and metabolic secretion patterns, particularly phenylpropanoids and isoprenoids, while AMF selectively enhanced cinnamic acids, sphingolipids, and hydroxycinnamic acids, suggesting a more targeted modulation of metabolic pathways.

Multi-omics data integration further confirmed strong correlations between root exudate composition and microbial community shifts, highlighting key metabolite biomarkers associated with biostimulant applications. Overall, these findings emphasize the potential of PGPR and AMF in mitigating the adverse effects of HM contamination by modulating root exudation patterns and influencing rhizosphere microbial communities. These results support the strategic use of MBs as sustainable tools for improving plant resilience and soil health in HM-contaminated environments, paving the way for future applications in phytoremediation and sustainable agriculture.

## Figures and Tables

**Table 1:** Relative variability and block contributions of the ANOVA multi-blocking orthogonal projection to latent structures discriminant analysis (AMOPLS) of tomato root exudates affected by heavy metal stress (HM), microbial biostimulant application (MB), and interaction between HM × MB. RSS: Relative sum of squares, Tp1–6: predictive components, To: orthogonal component.

Effect Name	RSS	RSS *p*-Value	R2Y *p*-Value	Tp1	Tp2	Tp3	Tp4	Tp5	Tp6	To1
HM	9.0%	0.01	0.01	1.8%	2.6%	94.4%	13.4%	73.0%	12.7%	26.2%
MB	23.2%	0.01	0.01	94.3%	1.7%	1.3%	57.7%	6.4%	8.5%	17.6%
HM × MB	14.4%	0.01	0.01	1.7%	92.5%	1.9%	12.8%	9.1%	63.7%	25.0%
Residuals	53.3%	-	-	2.2%	3.1%	2.4%	16.0%	11.4%	15.1%	31.2%

## Data Availability

The original contributions presented in this study are included in the article/Supplementary Material. Further inquiries can be directed to the corresponding author(s).

## Supplementary Materials

The following supporting information can be downloaded at: https://www.mdpi.com/article/10.3390/plants14213353/s1, Figure S1: HCA and AMOPLS score plot; Figure S2: HCA of Cd and Zn treatment groups; Figure S3: OPLS-DA models; Figure S4: bar plots of terpenoids and fatty acid classes in root exudates under Cd- and Zn-induced stress; Figure S5: box plot of IAAs; Figure S6: Loading plots from DIABLO model for Cd and Zn stress; Table S1: Exudate profiling dataset; Table S2: VIP markers related to Cd-inducted stress analysis; Table S3: VIP markers related to Zn-inducted stress analysis; Table S4: Loading features of DIABLO model for Cd-induced stress model; Table S5: Loading features of DIABLO model for Zn-induced stress model; Table S6: Network analysis for Cd-inducted stress model; Table S7: Network analysis for Zn-inducted stress model. References [50,51,52,53] are included in the Supplementary File.
