# Supplementary material for: Effects of Arbuscular Mycorrhizal Fungi and Metal-Tolerant Pseudomonas fluorescens on Mitigating Cadmium and Zinc Stress in Tomato
Source: Plants (Basel). 2025 Oct 31;14(21):3353. doi: 10.3390/plants14213353 (PMC12611042; doi:10.3390/plants14213353)
Supplement: Supplementary file 1 [file plants-14-03353-s001.zip › plants-3851958_supplementary_rev1/02_Supplementary Materials_revR1.docx]

**Effects of Arbuscular Mycorrhizal Fungi and Metal-Tolerant Pseudomonas fluorescens on Mitigating Cadmium and Zinc Stress in Tomato**

Leilei Zhang^1†*^, Gabriele Bellotti^1†^, Hajar Salehi^1^, Edoardo Puglisi^1^, Luigi Lucini^1,2^

*1 Department for Sustainable Food Process, Università Cattolica del Sacro Cuore, Piacenza, Italy*

*2 Institute of Bioimaging and Complex Biological Systems (IBSBC), National Research Council (CNR), Milan, Italy*

* Corresponding author: leilei.zhang@unicatt.it

† These authors contributed equally

**Supplementary Figures**

**
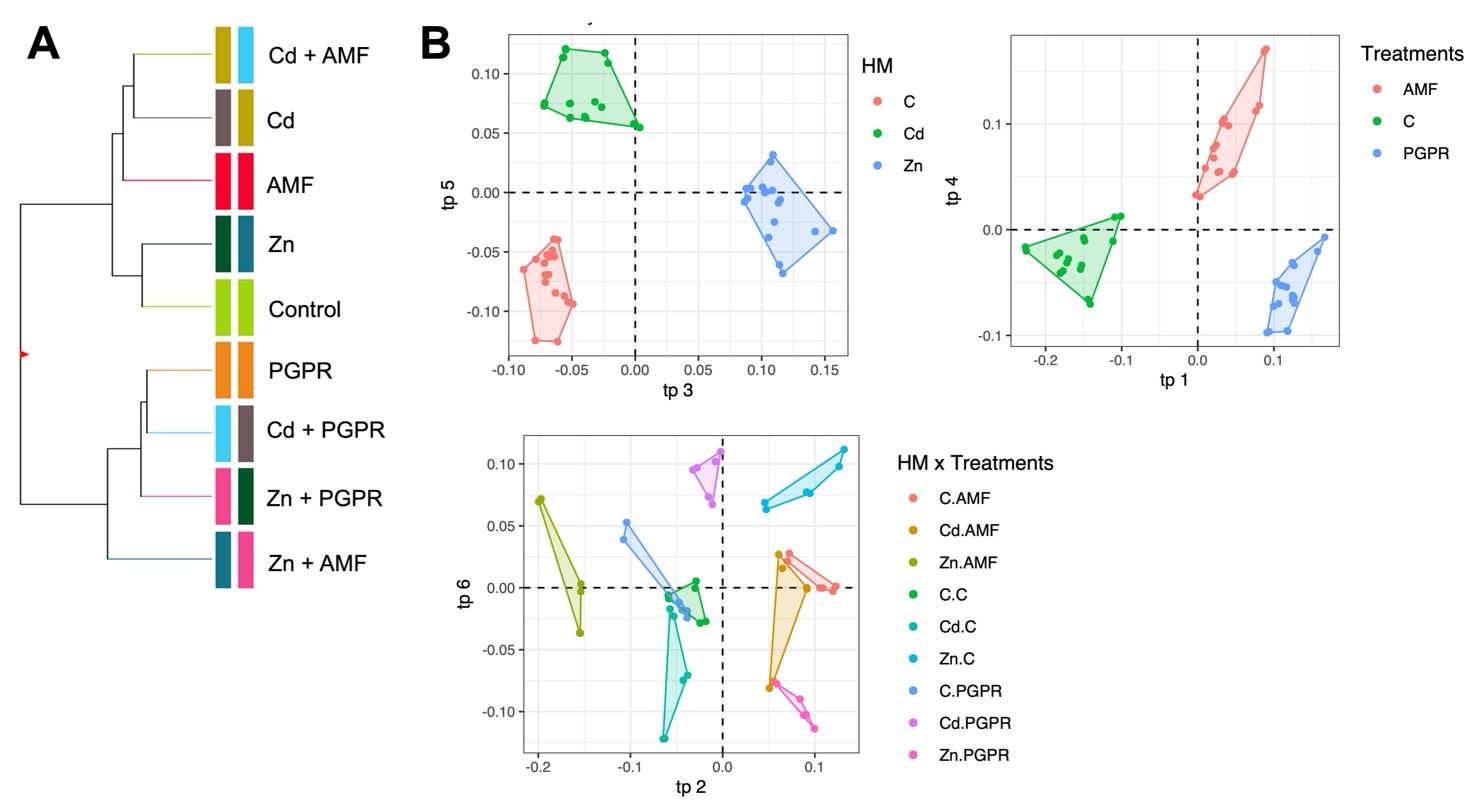
**

**Figure S1.** (A) Unsupervised hierarchical cluster analysis and (B) ANOVA multi-blocking orthogonal projection to latent structures discriminant analysis (AMOPLS) score plots of tomato root exudates affected by heavy metal stress (HM), microbial biostimulant application (MB), and interaction between HM x MB. Abbreviation: AMF = Arbuscular Mycorrhizae Fungi, PGPR = Plant Growth Promoting Rhizobacteria, C = Control, HM = Heavy Metal.


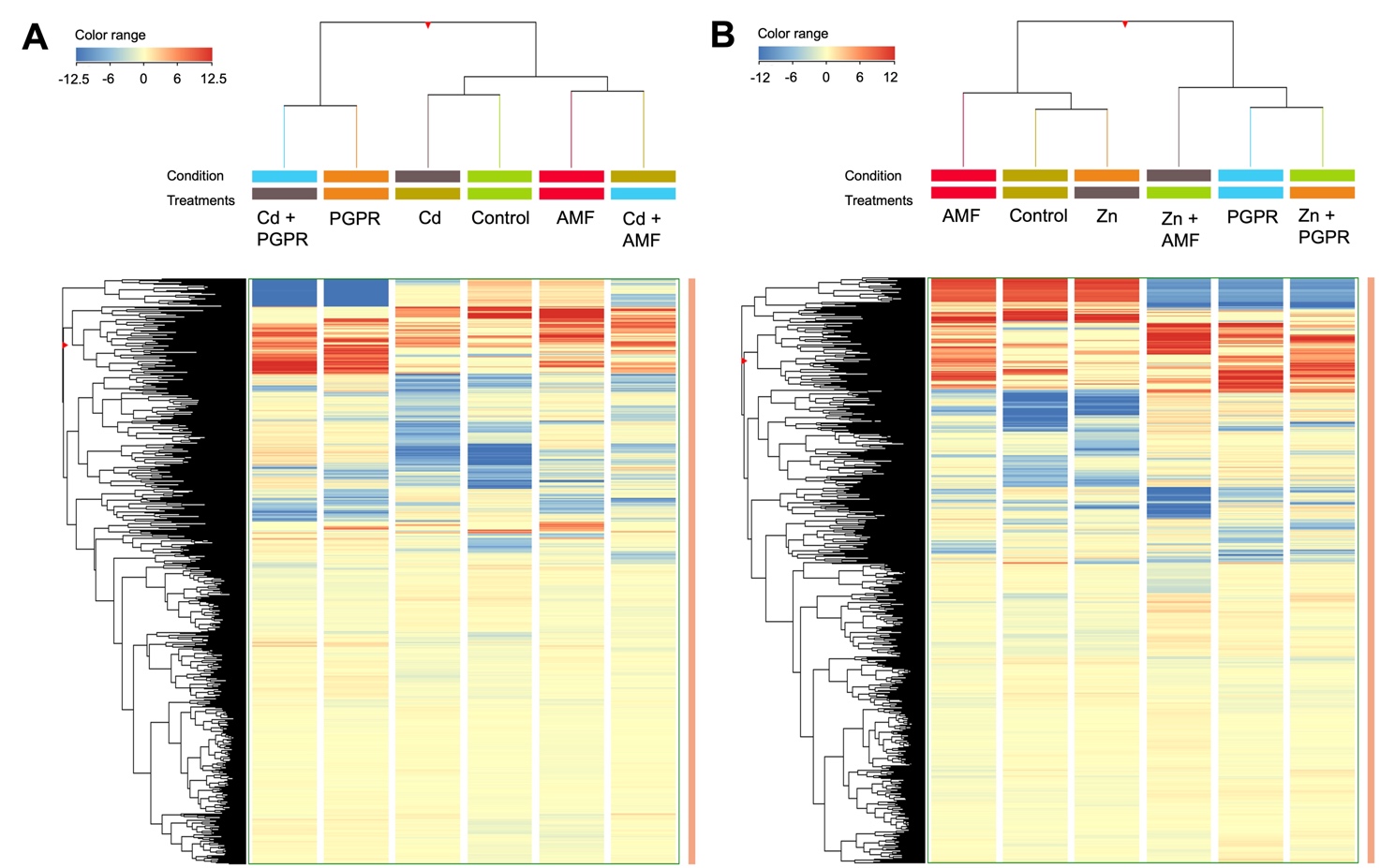


**Figure S2.** Unsupervised hierarchical cluster analysis of tomato root exudates for (A) Cd-stressed and (B) Zn-stressed plants. Abbreviation: AMF = Arbuscular Mycorrhizae Fungi, PGPR = Plant Growth Promoting Rhizobacteria.

**
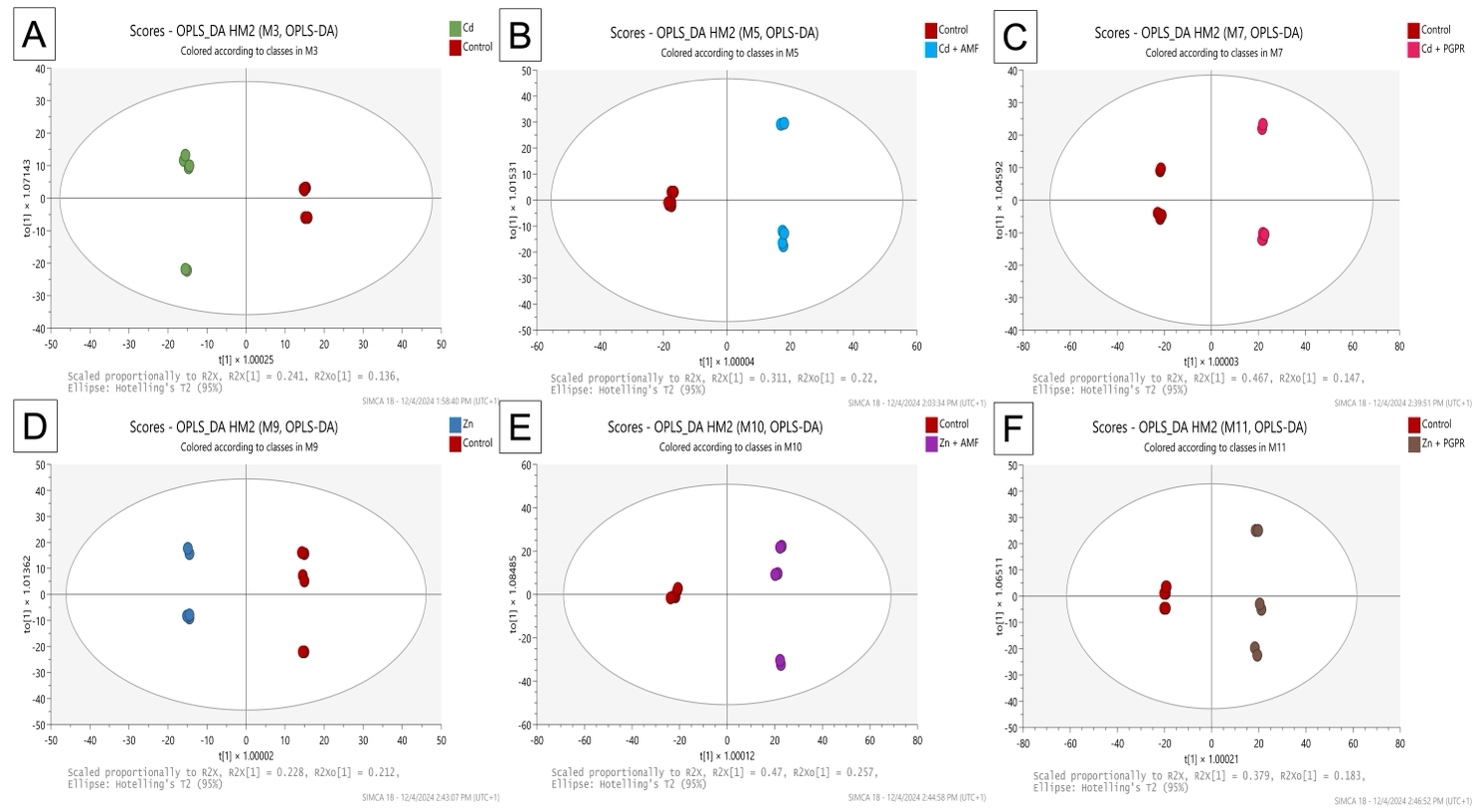
**

**Figure S3.** Orthogonal Partial Least Squares-Discriminant Analysis (OPLS-DA) models for (A-C) Cd-stressed and (D-F) Zn-stressed tomato plants.

**
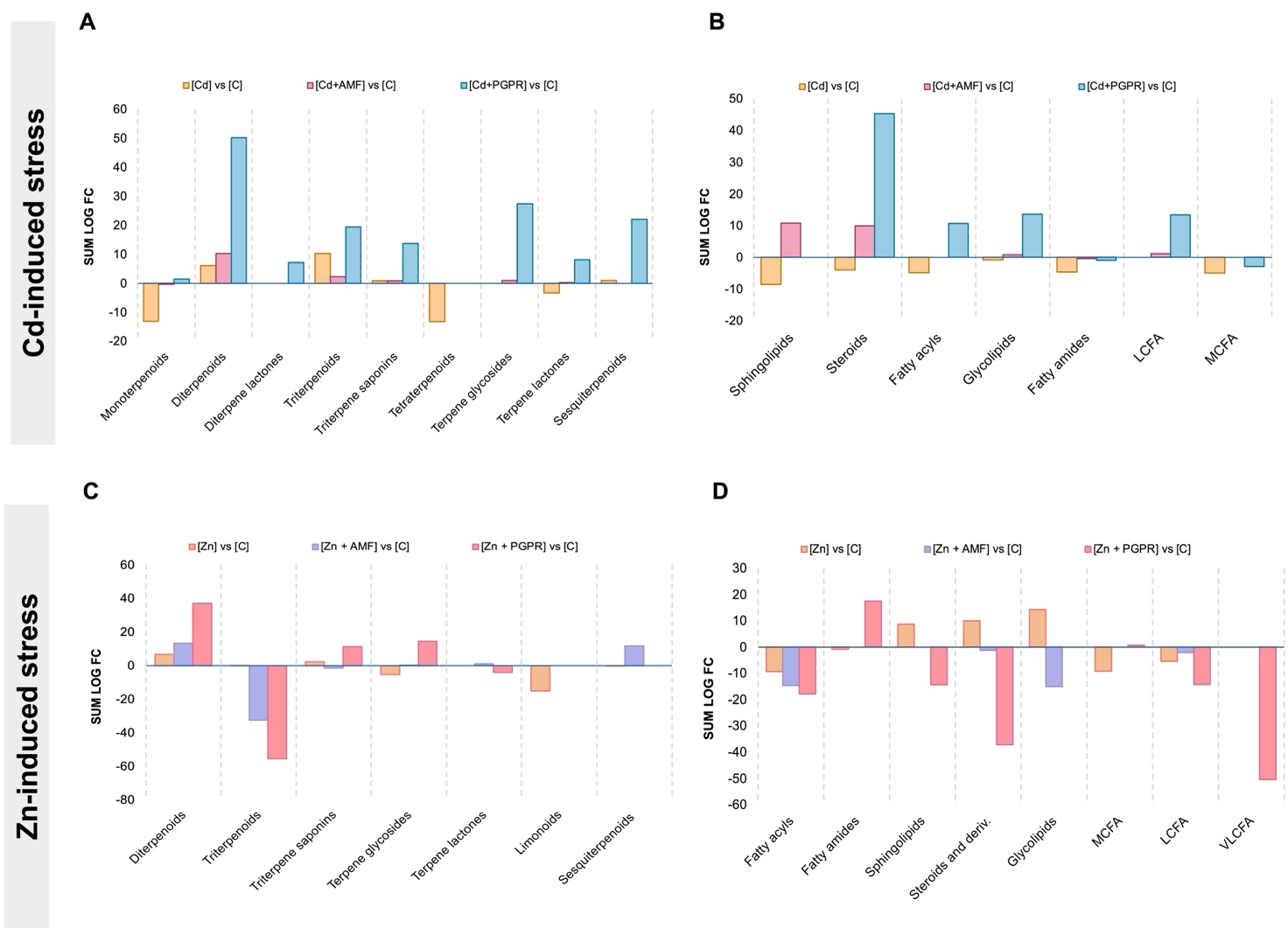
**

**Figure S4.** The unique compounds derived from Venn analysis reported in Figure 2 of the main manuscript were classified in their compound ontologies and represented in bar plots for the [A and C] terpenoids [B and D] fatty acids, as Cd and Zn-induced stress, respectively. Abbreviation: AMF = Arbuscular Mycorrhizae Fungi, PGPR = Plant Growth Promoting Rhizobacteria, C = Control.

**Figure S5.** Box plot of Indole-3-acetic acid (IAA) abundances across different treatments.


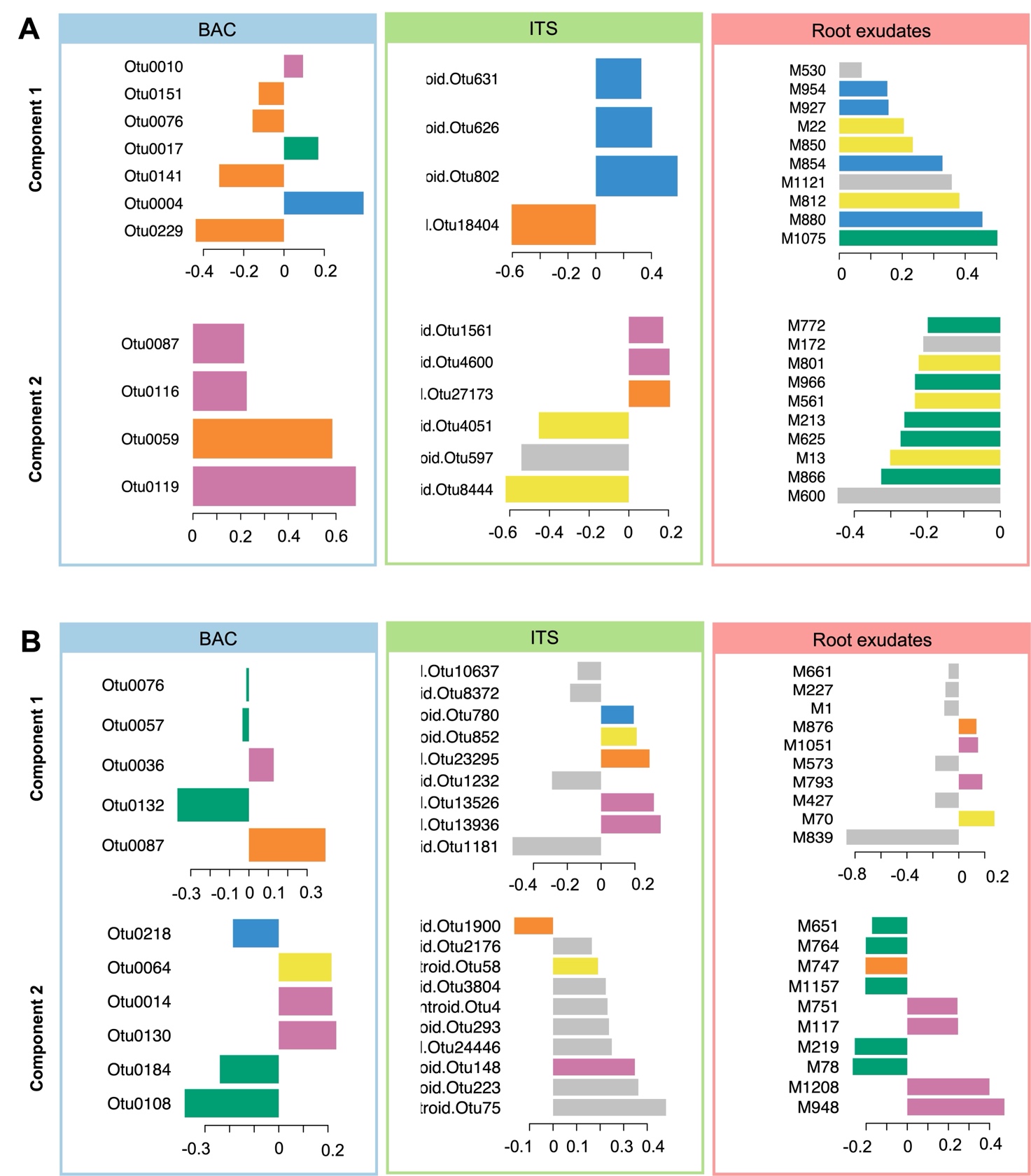


Figure S6. DIABLO-based data integration models for the exudate dataset and rhizosphere bacterial and fungal metagenomics datasets of soil and root system of the tomato plant. Loading plots of the first two components for the three blocks for [A] Cd and [B] Zn stress models. The corresponding feature codes are listed in Supplementary Table S5. Abbreviation: AMF = Arbuscular Mycorrhizae Fungi, PGPR = Plant Growth Promoting Rhizobacteria, C = Control.

**Supplementary materials and methods**

1. **Exudate profiling methodology**

The root exudate metabolites were profiled through the 6560-drift tube-ion mobility-quadrupole-time of flight-high resolution mass spectrometer (DTIM-UHPLC-QTOF-HRMS; Agilent Technologies, Santa Clara, CA, USA). The chromatographic separation was achieved under a water-acetonitrile (both LC-MS grade, from Sigma-Aldrich, Milan, Italy) gradient elution (6–94 % acetonitrile in 32 min), flow rate of 0.2 mL/min and injection volume of 6 μL, using 0.1 % (v/v) formic acid as phase modifier on an Agilent Zorbax Eclipse plus C18 analytical column (50 × 2.1 mm, 1.8 μm). The QTOF mass analyzer operated in positive mode (ESI+) for both MS and MS/MS acquisition with nitrogen as both sheath gas (12 L/min and 315 ◦C) and drying gas (14 L/min and 250 ◦C). The nebulizer pressure was 45 psi, the nozzle voltage was 350 V, and the capillary voltage was 4.0 kV. For MS acquisition, the full scan mode was performed within the range of m/z 100–1200 (1 spectra/s), mass resolution of 30,000 full width at half maximum (FWHM), m/z = 200. The data-dependent mode was performed for precursor fragmentation (10, 20, and 40 eV) and acquisition of MS/MS data from QC samples, with a mass resolution of 30,000 (FWHM), selecting 8 precursors per cycle (1 Hz, m/z 80–1200, positive polarity, and active exclusion after 2 spectra). The collected data (.d files) were processed using MS-DIAL software (version 4.70) for automatic peak finding, LOWESS normalization, and annotation via spectral matching, exploiting the comprehensive BMDMS-NP, Fiehn/Vaniya natural product library, and GNPS databases. The mass range 100–1200 m/z was searched for features with a minimum peak height of 10,000 cps, using an accurate mass tolerance for peak centroiding of 0.05 and 0.1 Da, for MS and MS/MS, respectively. Retention time information was excluded from the calculation of the total score. The identification step was based on mass accuracy, isotopic pattern, and spectral matching. These criteria were used to calculate a total identification score, using a minimum cut-off value of 70 %, considering the most common HESI + adducts.

1. **Amplicon sequencing methodology**

Total DNA was extracted from 500 mg of rhizosphere with the FastDNA™ SPIN Kit for Soil (MPBio, USA). Yields were measured with the Quant-iT™ HS ds-DNA assay kit (Invitrogen, Paisley, United Kingdom) using a QuBit™ fluorometer. To assess the bacterial and fungal population in each sample a nested PCR with barcoded forward primers was set up as described in (Bellotti et al., 2022) using the universal primers 343f (5′-TACGGRAGGCAGCAG-3′), and 802r (5′-TACNVGGGTWTCTAATCC-3′ for bacteria, and ITS-1 (5′- TCCGTAGGTGAACCTGCGG-3′), and ITS-2 (5’-GCTGCGTTCTTCATCGATGC-3′ for fungi. For each sample 30 ng of PCR product (V3-V4 or ITS1) were pooled and purified with the solid phase reversible immobilization (SPRI) method Agencourt AMPure XP kit (Beckman Coulter, Italy) following the manufacturer’s instruction. High-throughput sequencing (HTS) was performed by Fasteris S.A. (Geneva Switzerland), using the TruSeq DNA sample preparation kit (Illumina Inc., San Diego, CA) for amplicon library preparation, with MiSeq Illumina instrument (Illumina Inc., San Diego, CA) generating 300 bp paired-end reads.

Raw reads processing began with barcode demultiplexing and base calling, performed using Illumina MiSeq Control Software version 2.3.0.3, RTA v1.18.42.0, and CASAVA v1.8.2. Raw reads were assembled into amplicon sequences through PANDAseq software (Masella et al., 2012), applying a minimum overlap of 30 bp between paired reads and permitting a maximum of two mismatches per sequence. Sequences were subsequently demultiplexed based on primer tag sequences using the Fastxtoolkit.1 software. To ensure data quality, chimeric sequences (homopolymers exceeding 10 bp) that failed to align with the target regions V3-V4 for bacteria and ITS1 for fungi were excluded. This filtering step utilized Mothur version 1.32.0 for bacterial sequences and the UCHIME algorithm with the UNITE database for fungal sequences.

The resulting high-quality sequences were analyzed using two distinct methods: the operational taxonomic unit (OTU) approach and the taxonomy-based approach. For the bacterial V3-V4 region, OTUs and taxonomic assignments were processed using Mothur v1.32.1 (Schloss et al., 2009). For ITS1 amplicons, OTUs were identified using UPARSE (Edgar, 2013), as no aligned reference databases exist for ITS1. Mothur was configured with a minimum sequence length of 120 bp, while no upper length limit was applied to accommodate the size variability typical of ITS sequences.

1. **Statistical Analysis**

Root exudate profile raw data were processed using Mass Profiler Professional 12.6 (Agilent Technologies). The raw data underwent log2 transformation, 75th percentile normalization, and baseline correction against each compound’s median. To identify the contribution in discrimination capacity of HM and microbial biostimulant application factors on the overall data variance, a supervised ANOVA Multiblock Orthogonal Partial Least Squares (AMOPLS) analysis was performed using the rAMOPLS package in R (version 4.2.1). Statistical significance for AMOPLS was set at α = 0.01 and validated through 100 permutation tests. Results were expressed as Relative Sum of Squares (RSS), indicating the percentage of variability attributed to each factor; RSS p-value, denoting statistical significance; and block contribution, representing the percentage associated with each effect. Subsequently, unsupervised hierarchical cluster analysis (HCA) was conducted to explore sample patterns, employing Euclidean distance and Ward's linkage method for clustering separately for Cd- and Zn- driving stress models.

To identify Variable Importance in Projection (VIP) markers associated with different treatments compared with the control (C) untreated sample, the supervised orthogonal projection to latent structures discriminant analysis (OPLS-DA), using the SIMCA software (v.16, Umetrics®, Malmö, Sweden), were carried out separately for [HM stress *vs*. C], [HM + AMF *vs*. C], and [HM + PGPR *vs*. C]. The quality of OPLS-DA models was further evaluated in terms of goodness-of-fit (R^2^Y) and goodness-of-prediction (Q^2^Y), being statistically validated through cross-validation analysis of variance (CV-ANOVA) and excluding the overfitting by permutation test (n = 100). Specifically, those metabolites showing the highest influence in the discrimination between treatments, assuming a VIP score threshold of 1.2, were selected for both Cd and Zn groups.

To better focus on the specific effects of MB applications under HM stress, a Venn diagram was applied to identify uniquely metabolites associated with each treatment: HM alone, HM + AMF, and HM+PGPR. Compared to the control, these unique metabolites were further analyzed in terms of fold change analysis. Finally, VIP markers linked to each treatment were categorized into metabolite classes. The results were visualized using bar plots that compare [HM stress vs. Control], [HM + AMF vs. Control], and [HM + PGPR vs. Control] for both Cd and Zn treatment groups.

Statistical evaluations HTS data were carried out using Mothur software alongside R v3.0.02, incorporating the Vegan package (Dixon, 2003). Further methodological specifics are detailed in Vasileiadis et al., 2015). Α-diversity indices such as Shannon’s Index, Observed Richness (S), Simpson’s Diversity Index (D), and Chao’s Index were utilized to examine variations in microbial communities corresponding to each treatment. Principal Component Analysis (PCA) was applied to investigate unconstrained groupings among samples, while Canonical Correspondence Analysis (CCA) was employed to evaluate the influence of different treatments on the observed diversity.

1. **Multi-omics data integration**

The DIABLO model was optimized using the framework's tuning function. This function identifies the ideal number of components by minimizing the balanced error rate related to centroid distances. This optimization revealed four components for the Cd model and three for the Zn model. The results for the optimized DIABLO model were visualized through block contribution plots for each dataset (“M” for exudates, “OTU” for bacteria, and “centroid.OTU” for fungi). Significant features contributing to the differentiation between treatments were identified for each component and recognized as key biomarkers. The highly correlated features (key biomarkers; r > |0.75|) deriving from the three blocks were used to build network analysis using the “igraph” R package. Finally, the significantly correlated OTUs were taxonomically identified by submitting OTU sequences to NCBI (National Center for Biotechnology Information). The taxonomy assignment used the BLAST function with default parameters, targeting the 16S rRNA database for bacterial sequences and the ITS database for fungal sequences.

References:

Bellotti, G., Taskin, E., Guerrieri, M.C., Beone, G.M., Menta, C., Remelli, S., Bandini, F., Tabaglio, V., Fiorini, A., Capra, F., Bortolaso, R., Sello, S., Sudiro, C., Cocconcelli, P.S., Vuolo, F., Puglisi, E., 2022. Agronomical valorization of eluates from the industrial production of microorganisms: Chemical, microbiological, and ecotoxicological assessment of a novel putative biostimulant. Front Plant Sci 13. https://doi.org/10.3389/fpls.2022.907349

Edgar, R.C., 2013. UPARSE: highly accurate OTU sequences from microbial amplicon reads. Nat Methods 10, 996–998. https://doi.org/10.1038/nmeth.2604

Masella, A.P., Bartram, A.K., Truszkowski, J.M., Brown, D.G., Neufeld, J.D., 2012. PANDAseq: Paired-end assembler for illumina sequences. BMC Bioinformatics 13. https://doi.org/10.1186/1471-2105-13-31

Schloss, P.D., Westcott, S.L., Ryabin, T., Hall, J.R., Hartmann, M., Hollister, E.B., Lesniewski, R.A., Oakley, B.B., Parks, D.H., Robinson, C.J., Sahl, J.W., Stres, B., Thallinger, G.G., Van Horn, D.J., Weber, C.F., 2009. Introducing mothur: Open-source, platform-independent, community-supported software for describing and comparing microbial communities. Appl Environ Microbiol 75, 7537–7541. https://doi.org/10.1128/AEM.01541-09
